# Paternal and maternal support of moderate-to-vigorous physical activity in children on weekdays and weekends: a cross-sectional study

**DOI:** 10.1186/s12889-021-11730-8

**Published:** 2021-09-30

**Authors:** Meijing An, Tianjiao Chen, Qianling Zhou, Jun Ma

**Affiliations:** 1 Institute of Child and Adolescent Health, School of Public Health, Peking University, No. 38 Xueyuan Road, Haidian District, Beijing, 100191 China; 2 Department of Maternal and Child Health, School of Public Health, Peking University, No. 38 Xueyuan Road, Haidian District, Beijing, 100191 China

**Keywords:** Father, Mother, Support behaviours, Moderate-to-vigorous physical activity, Children

## Abstract

**Background:**

Most studies of associations between parental support behaviours for physical activity (PA) and children’s moderate-to-vigorous physical activity (MVPA) have been conducted in developed countries, and they have focused on maternal or parental support behaviours. Children’s MVPA time (i.e., weekdays vs. weekends) has not been adequately differentiated. This paper investigated the associations of paternal and maternal support behaviours for PA with the proportion of children who met the MVPA recommendations on weekdays and weekends in China.

**Methods:**

Cross-sectional data of 517 father–child dyads and 1422 mother–child dyads were analysed. The children recorded PA diaries on 7 consecutive days to assess their MVPA time. The father or mother completed a questionnaire concerning their support for children’s PA. Multivariate logistic regression was used to investigate the independent effects of paternal and maternal support behaviours for PA on whether children met the MVPA recommendations for weekdays, weekends, and the whole week.

**Results:**

Significantly fewer children met the MVPA recommendations on weekends (37.8%) than on weekdays (62.8%). Higher paternal (odds ratio [OR] = 1.098, 95% confidence interval [CI]: 1.009–1.195) and maternal (OR = 1.076, 95% CI: 1.021–1.134) total support behaviour scores were associated with higher odds of children meeting the MVPA recommendations on weekends, after controlling for covariates. Paternal PA knowledge-sharing with the child was positively associated with children meeting the MVPA recommendations on weekends (OR = 1.319, 95% CI: 1.055–1.649); it tended to be associated with children meeting the MVPA recommendations on weekdays and throughout the week, although these associations were not statistically significant. Maternal reservation of PA time for the child was positively associated with a higher likelihood of children meeting the MVPA recommendations on weekdays (OR = 1.160, 95% CI: 1.025–1.313), weekends (OR = 1.241, 95% CI: 1.097–1.403), and throughout the week (OR = 1.214, 95% CI: 1.076–1.369).

**Conclusions:**

Paternal and maternal support behaviours for PA should be enhanced on weekends to increase children’s MVPA. Fathers should share PA knowledge with children and mothers should reserve PA time for children every day.

**Supplementary Information:**

The online version contains supplementary material available at 10.1186/s12889-021-11730-8.

## Background

Physical activity (PA), especially moderate-to-vigorous physical activity (MVPA), improves children’s health [1–4]. MVPA reduces the risks of obesity [1] and diabetes [2]; it enhances bone [3] and mental [4] health in children aged 5 to 17 years. The World Health Organisation [5] recommends that children should have a mean of at least 60 min per day of moderate-to-vigorous (mostly aerobic) PA throughout the week. This recommendation is similar to relevant guidelines in China [6], the United States [7], Canada [8], and Australia [9]. However, many children worldwide do not meet this recommendation [10–12]. Only 20.1% of children aged 9 to 15 years in Japan [10] and 26.2% of children aged 6 to 17 years in Thailand [11] achieved the MVPA recommendation. In 2013, Wang et al. reported that only 3.7–14.0% of boys and 0.7–3.0% of girls aged 9 to 17 years in China met the MVPA recommendation [12]. Regardless of age group, children performed fewer minutes of MVPA per day on weekends than on weekdays [12]. A meta-analysis showed that among children aged 4 to 18 years, the mean difference in MVPA between weekdays and weekends was 0.42 min [13]. Some modifiable factors improved children’s PA and healthy growth, such as self-efficacy, family support, social support, walkability, and access to recreation facilities [14].

Bauman et al. used an ecological model to identify the correlates or determinants of PA among children aged 5 to 18 years using demographic/biological, psychosocial, behavioural, social and cultural, and environmental factors [14]. Within the social and cultural classification, parents’ or guardians’ support (e.g., encouragement and financial, instrumental, and emotional support) for PA was positively associated with children’s PA [14–18]. A meta-analysis published in 2015 showed that the association between parental support and children’s PA or MVPA was moderate (*r* = 0.38, 95% confidence interval [CI]: 0.30–0.46) and parental encouragement was moderately associated with children’s PA (*r* = 0.34, 95% CI: 0.25–0.41). The meta-analysis included studies using accelerometers, activity monitors, children’s self-report data, and parents’ reported data in minutes, hours, or days per week to measure MVPA [19]. Recent studies reported similar findings [15, 17, 20]. A large cross-sectional study (*n* = 81,857) in China showed that parental encouragement and financial support for PA were positively associated with a high level of MVPA among students aged 9–17 years [15]. In the US, parental instrumental and emotional support for PA were significantly associated with the times per week children engaged in PA [17], and parental support for PA was positively associated with children’s minutes per hour of MVPA [20]. In most studies, mothers were the main respondents [19], while some studies included fathers or reported paternal data separately [21]. Currently, men are involved more in childcare than in the past [22, 23]. It is becoming more important to understand fathers’ roles in improving children’s MVPA participation because the association between paternal support for PA and children’s MVPA remains unclear.

Some studies reported differences between paternal and maternal support behaviours for PA [24, 25]. While mothers tended to play a larger role in logistical support of PA, fathers were more likely than mothers to engage in modelling PA for their daughters [24]. A study of Japanese boys (9.4 ± 1.6 years) and girls (9.4 ± 1.6 years) from grades one to six demonstrated that maternal support behaviour, such as watching their children’s sporting events, was associated with a high level of children’s MVPA; however, it failed to find an association between paternal support and children’s MVPA [26]. An Australian study showed that maternal role-modelling or paternal reinforcement of PA was positively associated with boys’ after school or weekend MVPA [27]. Most studies of parental support behaviours were conducted in developed countries, such as Japan and Australia [19]. The associations of paternal or maternal support behaviours with children’s MVPA were inconsistent across countries [26, 27].

Thus far, only a few studies have examined parental support behaviours for children’s MVPA at different times (i.e., weekdays vs. weekends). A German study found that increased family support resulted in an increased total MVPA time and MVPA time after 3 pm on weekdays [28]. An Australian study found that different support activities by the mother (role modelling) or father (reinforcing PA) were positively associated with boys’ mean minutes per day of MVPA after school or on weekends [27]. A Canadian study used pedometers to assess PA in steps per day among grade five children (mean age, 10.9 ± 0.4 years) and found that increased parental encouragement was positively associated with PA among boys and girls on weekdays and PA among girls on weekends. Increased parental care (i.e., very much vs. quite a lot) was positively associated with PA among boys on weekends measured using pedometer [29]. Differentiating times (i.e., weekdays vs. weekends) might be useful for developing interventions or strategies to promote children’s MVPA. In China, one study reported that parental support was associated with children’s MVPA for the whole week, without differentiating between children’s MVPA on weekdays or weekends [15].

Parental support has important roles in establishing and shaping children’s MVPA habits [15, 16, 19]; however, there are gaps to be explored. First, little is known regarding paternal PA support towards school-aged children, especially in China. Second, parental support behaviours for PA on children’s MVPA at different times (i.e., weekdays vs. weekends) have not been studied adequately in China or elsewhere. Therefore, this study investigated the associations of paternal and maternal support for PA with the proportion of children who met MVPA recommendations, differentiating between weekdays and weekends. We hypothesised that paternal and maternal support behaviours for PA would be positively associated with the proportion of children meeting MVPA recommendations and that there would be different associations on weekdays and weekends.

## Methods

### Participants

This cross-sectional study using stratified cluster sampling was conducted between October and December 2012 in Fangshan District, Beijing, China. Students (7–15 years old) from grades two to five and seven to eight, along with their caregivers (i.e., the primary person caring for the child’s daily life outside school), were recruited from 16 schools (*n* = 4 each: urban middle, rural middle, urban primary, and rural primary). Students with major organic diseases (e.g., heart, lung, liver, kidney, and related diseases), abnormal body development, or cause-based obesity (e.g., endocrine disease or drug side effect) were excluded. Approval was obtained from the Ethics Committee of Peking University. Written informed consent was obtained from all participating children and their parents before any assessment.

Seven-day PA diaries were provided to 3441 students and 3441 questionnaires were administered to caregivers. When the head teacher collected the student’s PA diaries, they checked the integrity of the PA diaries. Any blanks in the PA diaries were required to be filled in on the spot. In all, 2810 seven-day PA diaries were collected. If data in the PA diary met the following four criteria, the PA diary was valid: the proportions of missing values were less than 30% for all variables, less than 10% for qualitative variables, and less than 30% for quantitative variables; the PA diary was completed carefully; there were no logical errors in the PA diaries; and a day with less than 30% missing values for all variables. The PA diary was valid if it had records for ≥3 valid weekdays and ≥ 1 weekend day. There were 2670 PA diaries considered as valid. There were 3430 parental questionnaires were collected. The parental questionnaires were then paired with children’s PA diaries. The data was further excluded if the respondents’ identity was unclear (*n* = 389), the parental questionnaire was not completed by the mother or father (*n* = 296), or ≥ 1 parental support behaviour was not filled in the caregiver’s questionnaire (*n* = 46). This yielded 1939 valid parental questionnaires: 517 completed by fathers and 1422 completed by mothers.

### Measures

#### MVPA

PA among children and adolescents was assessed using 7-consecutive-day PA diaries, which were developed specifically for this study (see Additional file 1). Students were asked to mark the activities and record the lengths of the activities close to bedtime every day. Parents could assist children in grade three and below who could not record their behaviour diaries independently. The diary was developed based on items in a Chinese 7-day recall PA questionnaire used by 92 primary school children (mean age, 10.5 ± 1.0 years) in Beijing, China [30]. The Chinese 7-day recall PA questionnaire had good content validity and face validity, acceptable criterion validity (r = 0.26 measured by the Caltrac measuring instrument, *P* < 0.05), and good test–retest reliability (Kappa values, 55.2–73.4%). Considering the children’s activities in the current study, questions concerning going to or leaving school, walking, and sedentary behaviours (e.g., watching television and playing on computers) were kept; behaviours regarding homework and physical education (PE) classes were added. Some activities were categorised into the MVPA activity group with in- and out-of-school examples. The items included (1) ‘How did you go to school?’ and (2) ‘How did you go home after school? ’ The answers for these two items were ‘no school’, ‘walked’, ‘cycled’, and ‘rode in a car or bus, etc’. Children who walked, cycled, or rode in a car or bus, etc. were required to record the trip duration. The other questions were: (3) ‘Did you walk outside today?’ (e.g., shopping, visiting relatives or friends, or going to a park); (4) ‘Did you do homework today?’; (5) ‘Did you watch television today?’; (6) ‘Did you play computer/iPad or e-games today?’; (7) ‘Did you have physical education (PE) class today?’; (8) ‘In addition to PE class, did you do MVPA in school today?’; and (9) ‘Did you do MVPA outside of school?’ In (8) and (9), MVPA referred to activities that made the child sweat, gasp, or feel a little tired or very tired (e.g.*,* running, playing football, cycling, and dancing). The answers for items (3) to (9) were ‘yes’ or ‘no’. For a ‘yes’ response, the time length was also required to be provided.

In this study, cycling to or from school, attending PE class, and participating in MVPA in and outside of school were regarded as MVPA. The durations of each activity were summed to yield the total MVPA time each day. The time in PE class was added into the total MVPA minutes, in accordance with the classification for Children’s Leisure Activities Study Survey questionnaire [31]. Because a study in Shanghai, China reported that the proportion of MVPA time in middle school PE class was 40.8% [32], we used 40.8% as an estimated proportion of MVPA time in PE for the sensitivity analysis.

Missing data were added using the following strategies. First, missing values for PE and PE time were input in accordance with the results of other students in the same class. Second, the variable of attending activity (except for going/leaving to school and PE) was marked ‘yes’ if the duration exceeded zero. Third, the mode and duration of transportation between home and school were input as the mean values for other weekdays. Fourth, missing values for the times spent going out, homework, watching television, playing computer/iPad or e-games, in-school MVPA, and out-of-school MVPA were filled using the mean values of two nearby point values for students of the same gender in the same class. The mean MVPA time on weekdays was defined as the total validated MVPA time from Monday to Friday, divided by the number of validated days. The mean MVPA time on weekend days was defined as the total validated MVPA time for Saturday and Sunday, divided by the number of validated days. The mean time for all days was defined as the total validated MVPA time from Monday to Sunday, divided by the number of validated days. The daily MVPA times on weekdays, weekends, and the whole week were subcategorised in accordance with the PA recommendations of the World Health Organisation [5] and China [6]. The cut-off value was 60 min. A daily MVPA time ≥ 60 min met the MVPA recommendations, while < 60 min did not meet recommendations.

#### Parental support for physical activity

Parental support behaviours were evaluated using the following three questions on a 5-point Likert scale from never (1) to always (5); the questions were completed by the mother or father. Q1: ‘As a parent, do you share your knowledge of PA/exercise with your child?’ (hereinafter, share PA knowledge with the child). Q2: ‘Do you tell your child and help your child cultivate good exercise habits after you requiring knowledge of PA/exercise?’ (hereinafter, cultivate the child’s PA habits). Q3: ‘Do you reserve time for your child for PA/exercise?’ (hereinafter, reserve PA time for the child). Cronbach’s alpha coefficients of PA support were 0.762 for fathers and 0.778 for mothers. The sum of these three variables was used as the total paternal or maternal score of support behaviours.

#### Covariates

Gender, grade, and school name were collected from the PA diary, and information regarding each child’s district and school stage was obtained. The child’s age was calculated from their birthdate and examination date. Trained postgraduates measured the children’s heights and weights using a portable stadiometer (model TZG, China) and lever-type weight scale (model RGT-140, China). Body mass index (kg/m^2^) was calculated as the weight (kg) divided by height (m) squared. Children’s weight status (thin, normal, overweight, or obese) was defined based on age- and gender-specific body mass index cut-offs, in accordance with the screening standards for overweight and obesity among school-aged children in China in 2018 [33].

Paternal or maternal age, education level, and height and weight were obtained from the questionnaire. Paternal or maternal body mass index was calculated as above. Paternal or maternal weight status (thin, normal, overweight, or obese) was classified in accordance with the Chinese criteria of weight for adults [34]. Family structure was assessed with the question ‘Who is with the child the most while the child is at home?’ The response categories included mother, father, stepmother, stepfather, grandparents, and others. Based on the responses, the family structure was classified into four categories: both parents (a mother and a father), single parent (a mother or father), reconstituted families (a mother/father and a stepfather/stepmother) and others (e.g., grandparents) [35, 36].

### Data analysis

Normally distributed variables are presented as means and standard deviations. Categorical data are summarised using frequencies and percentages. Differences in proportions were examined by the chi-squared test for independent samples or McNemar’s test for matched samples. Differences in means for continuous variables were compared using Student’s *t*-test. Multivariate logistic regression was used to examine the associations between paternal or maternal support behaviour score and whether children met the MVPA recommendations on weekdays, on weekends, and for the whole week. Logistic regression was conducted after controlling for children’s school stage, gender, district, weight status, and paternal or maternal education level. All analyses were performed with SPSS Statistics (ver. 22.0). *P* < 0.05 was considered statistically significant.

## Results

Table 1 summarises the children’s and parents’ characteristics. The mean age was 11.35 ± 2.17 years. Overall, 34.6% of the children were overweight or obese. Overall, 57.0% of children met the MVPA recommendations for the whole week, while the proportions for weekdays (62.8%) and weekends (37.8%) significantly differed (*P* < 0.001). The children in father–child and mother–child dyads were similar in terms of age, schooling stage, weight status, and daily weekday, weekend, and total MVPA. The mean paternal and maternal ages were 38.83 ± 4.29 and 36.85 ± 4.55 years, respectively. The proportion of mothers ≥40 years was higher in father–child dyads (29.9%) than in mother–child dyads (24.5%) (Table 1). The proportion of maternal education of junior middle school or below was higher in father–child dyads (58.5%) than in mother–child dyads (47.4%). The parents in father–child and mother–child dyads were similar in terms of paternal age, paternal education level, paternal weight status, maternal weight status, and family structure.

**Table 1:** Characteristics of children and parents

Variables	All children (*n* = 1939)	Children in father-child dyads (*n* = 517)	Children in mother-child dyads (*n* = 1422)
*Children*			
Age (years), mean (SD)	11.35 ± 2.17	11.41 ± 2.13	11.32 ± 2.18
Male sex^a^,n(%)	959 (49.5)	288 (55.7)	671 (47.2)
Urban district^a^,n(%)	895 (46.2)	202 (39.1)	693 (48.7)
Primary school stage,n(%)	934 (48.2)	250 (48.4)	684 (48.1)
Overweight or obesity,n(%)	670 (34.6)	167 (32.3)	503 (35.4)
Daily weekday MVPA≥60 min,n(%)	1218 (62.8)	325 (62.9)	893 (62.8)
Daily weekend MVPA≥60 min,n(%)	732 (37.8)	196 (37.9)	536 (37.7)
Daily MVPA≥60 min,n(%)	1106 (57.0)	303 (58.6)	803 (56.5)
*Parents*			
Paternal age*,≥40 years old	688 (39.9)	197 (41.7)	491 (39.2)
Maternal age*^,a^,≥40 years old	447 (25.9)	134 (29.9)	313 (24.5)
Paternal education level*			
Junior middle school or below	862 (49.9)	254 (53.8)	608 (48.5)
Senior middle school or junior college	688 (39.9)	179 (37.9)	509 (40.6)
University or above	176 (10.2)	39 (8.3)	137 (10.9)
Maternal education level*^,a^			
Junior middle school or below	867 (50.3)	262 (58.5)	605 (47.4)
Senior middle school or junior college	671 (38.9)	148 (33.0)	523 (41.0)
University or above	186 (10.8)	38 (8.5)	148 (11.6)
Paternal overweight or obesity*	1100 (63.7)	291 (61.7)	809 (64.5)
Maternal overweight or obesity*	749 (43.4)	183 (40.8)	566 (44.4)
Family structure*			
Both parents	1675 (86.9)	442 (85.8)	1233 (87.3)
Single parent	180 (9.3)	50 (9.7)	130 (9.2)
Reconstituted families	16 (0.8)	2 (0.4)	14 (1.0)
Others	57 (3.0)	21 (4.1)	36 (2.5)

*Note*. *Variables existed missing values. ^a^Chi-square test of significance between children in father-child dyads and children in mother-child dyads (*P* < 0.05)

As shown in Table 2, paternal total support scores were higher than maternal total support scores. The distributions of the three support behaviours (i.e., sharing PA knowledge with the child, cultivating the child’s PA habits, and reserving PA time for the child) were similar among fathers and mothers.

**Table 2:** Support behaviors for PA of father and mother

Variables	Father	Mother
Total support behaviour scores^a^	10.21 ± 2.28	9.93 ± 2.21
Share PA knowledge with the child		
Never	5 (1.0)	15 (1.1)
Rarely	59 (11.4)	155 (10.9)
Sometime	185 (35.8)	615 (43.2)
Often	205 (39.6)	494 (34.7)
Always	63 (12.2)	143 (10.1)
Cultivate the child’s PA habits		
Never	4 (0.8)	10 (0.7)
Rarely	71 (13.7)	160 (11.3)
Sometime	179 (34.6)	583 (41.0)
Often	201 (38.9)	517 (36.3)
Always	62 (12.0)	152 (10.7)
Reserve PA time for the child		
Never	14 (2.7)	33 (2.3)
Rarely	108 (20.9)	387 (27.2)
Sometime	197 (38.1)	562 (39.5)
Often	145 (28.0)	338 (23.8)
Always	53 (10.3)	102 (7.2)

*Note*. This table provides frequency and percentage for categorical variables, and provides means and standard deviations for continuous variables. ^a^Student’s t-test of significance between father and mother (*P* < 0.05)

Children from rural districts were more likely to meet the MVPA recommendations on weekdays and for the whole week, compared with children from urban districts. Primary school students were less likely to meet the MVPA recommendations on weekdays, but more likely to meet recommendations on weekends, compared with middle school students. Normal weight or thin students were more likely to meet the MVPA recommendations on weekdays, compared with their overweight or obese counterparts. Paternal and maternal education level and maternal weight status were both associated with children’s MVPA on weekdays (see Additional file 2, Table S1).

Tables 3 and 4 show the respective univariate associations of paternal and maternal support behaviours with children’s MVPA. Paternal total scores for support behaviours and sharing PA knowledge with the child were higher in children who met the MVPA recommendations on weekends than in children who did not meet them (total support score: 10.48 vs. 10.03, *P* = 0.029; sharing PA knowledge with the child: 3.62 vs. 3.44, *P* = 0.026). Maternal total scores of support behaviours were higher in children who met the MVPA recommendations on weekends and throughout the whole week (weekends: 10.15 vs. 9.80, *P* = 0.004; whole week: 10.06 vs. 9.77, *P* = 0.015). The maternal score for reserving PA time for children was positively associated with children meeting the MVPA recommendations on weekdays, weekends, and throughout the week (weekdays: 3.12 vs. 2.97, *P* = 0.005; weekends: 3.19 vs. 2.99, *P* < 0.001; whole week: 3.14 vs. 2.96, *P* < 0.001).

**Table 3:** Univariate association between paternal support behaviors and children’s MVPA

Variables	Daily weekday MVPA time		Daily weekend MVPA time		Daily MVPA time	
	<60 min (*n* = 192)	≥60 min (*n* = 325)	<60 min (*n* = 321)	≥60 min (*n* = 196)	<60 min (*n* = 214)	≥60 min (*n* = 303)
Total support behaviour scores	10.10 ± 2.28	10.26 ± 2.28	**10.03 ± 2.29**	**10.48 ± 2.24**	10.07 ± 2.31	10.30 ± 2.26
Share PA knowledge with the child	3.46 ± 0.90	3.53 ± 0.87	**3.44 ± 0.89**	**3.62 ± 0.87**	3.46 ± 0.91	3.54 ± 0.86
Cultivate the child’s PA habits	3.46 ± 0.92	3.49 ± 0.89	3.42 ± 0.90	3.57 ± 0.90	3.46 ± 0.93	3.49 ± 0.88
Reserve PA time for the child	3.18 ± 1.03	3.25 ± 0.95	3.17 ± 0.99	3.30 ± 0.95	3.15 ± 1.01	3.27 ± 0.96

*Note*. Bold results refer to significant difference between group ≥60 min and <60 min in each time interval (*P* < 0.05)

**Table 4:** Univariate association between maternal support behaviors and children’s MVPA

Variables	Daily weekday MVPA time		Daily weekend MVPA time		Daily MVPA time	
	<60 min (*n* = 529)	≥60 min (*n* = 893)	<60 min (*n* = 886)	≥60 min (*n* = 536)	<60 min (*n* = 619)	≥60 min (*n* = 803)
Total support behaviour scores	9.80 ± 2.16	10.01 ± 2.23	**9.80 ± 2.18**	**10.15 ± 2.24**	**9.77 ± 2.15**	**10.06 ± 2.24**
Share PA knowledge with the child	3.40 ± 0.84	3.43 ± 0.86	3.40 ± 0.84	3.44 ± 0.87	3.39 ± 0.85	3.44 ± 0.86
Cultivate the child’s PA habits	3.43 ± 0.84	3.47 ± 0.86	**3.41 ± 0.85**	**3.52 ± 0.86**	3.42 ± 0.83	3.48 ± 0.87
Reserve PA time for the child	**2.97 ± 0.91**	**3.12 ± 0.95**	**2.99 ± 0.94**	**3.19 ± 0.93**	**2.96 ± 0.93**	**3.14 ± 0.94**

*Note*. Bold results refer to significant difference between group ≥60 min and <60 min in each time interval (*P* < 0.05)

Tables 5 and 6 show the respective adjusted associations of paternal and maternal support behaviours with children’s MVPA. After controlling for covariates, a higher paternal total support score was positively associated with children meeting the MVPA recommendations on weekends (odds ratio [OR] = 1.098, 95% CI: 1.009–1.195, *P* = 0.031; Table 5). Paternal PA knowledge-sharing with the child was positively associated with children meeting the MVPA recommendations on weekends (OR = 1.319, 95% CI: 1.055–1.649, *P* = 0.015); it tended to be associated with meeting MVPA recommendations on weekdays (OR = 1.220, 95% CI: 0.974–1.528, *P* = 0.084) and throughout the week (OR = 1.218, 95% CI: 0.977–1.519, *P* = 0.080), although these associations were not statistically significant. Similarly, maternal total support score was positively associated with meeting the MVPA recommendations on weekends (OR = 1.076, 95% CI: 1.021–1.134, *P* = 0.006, Table 6). Maternal cultivation of the child’s PA habits (OR = 1.171, 95% CI: 1.024–1.339, *P* = 0.021) was positively associated with meeting the MVPA recommendations on weekends. Maternal reservation of PA time for the child was positively associated with a higher likelihood of children meeting the MVPA recommendations on weekdays (OR = 1.160, 95% CI: 1.025–1.313, *P* = 0.018), weekends (OR = 1.241, 95% CI: 1.097–1.430, *P* = 0.001), and throughout the week (OR = 1.214, 95% CI: 1.076–1.369, *P* = 0.002).

**Table 5:** Adjusted association of paternal support behaviors and children’s MVPA meeting the recommendation

Variables	Daily weekday MVPA time	Daily weekend MVPA time	Daily MVPA time
Total support behaviour scores	1.053 (0.967,1.146)	**1.098 (1.009,1.195)**	1.061 (0.976,1.154)
Share PA knowledge with the child	1.220 (0.974,1.528)	**1.319 (1.055,1.649)**	1.218 (0.977,1.519)
Cultivate the child’s PA habits	1.050 (0.845,1.306)	1.213 (0.978,1.504)	1.031 (0.833,1.276)
Reserve PA time for the child	1.084 (0.892,1.318)	1.131 (0.933,1.372)	1.149 (0.948,1.392)

*Note.* In each logistic regression, outcome variable is 1 = < 60 min and 2 = ≥60 min. This table provides OR and 95%CI formatted as “OR (lower CI, upper CI)” for each result of logistic regression. Bold results indicate statistically significant (*P* < 0.05). Children’s school stage, gender, district, weight status, and paternal educational level were adjusted in each logistic regression

**Table 6:** Adjusted association of maternal support behaviors and children’s MVPA meeting the recommendation

Variables	Daily weekday MVPA time	Daily weekend MVPA time	Daily MVPA time
Total support behaviour scores	1.042 (0.989,1.098)	**1.076 (1.021,1.134)**	**1.060 (1.007,1.115)**
Share PA knowledge with the child	1.067 (0.929,1.224)	1.069 (0.932,1.226)	1.080 (0.944,1.235)
Cultivate the child’s PA habits	1.032 (0.903,1.180)	**1.171 (1.024,1.339)**	1.078 (0.946,1.228)
Reserve PA time for the child	**1.160 (1.025,1.313)**	**1.241 (1.097,1.403)**	**1.214 (1.076,1.369)**

*Note.* In each logistic regression, outcome variable is 1 = < 60 min and 2 = ≥60 min. This table provides OR and 95%CI formatted as “OR (lower CI, upper CI)” for each result of logistic regression. Bold results indicate statistically significant (*P* < 0.05). Children’s school stage, gender, district, weight status, and maternal educational level were adjusted in each logistic regression

In the sensitivity analysis, the proportions of children who met MVPA on weekdays, weekends, and throughout the week were lower than in the original analysis. The differences in the proportions of children who met the MVPA recommendations on weekdays (49.5%) and weekends (36.3%) were statistically significant (see Additional file 2, Table S2), which was similar to findings in the original analysis. The univariate associations of paternal or maternal support behaviours with children’s MVPA (see Additional file 2, Tables S3 and S4) were similar to findings in the original analyses. The adjusted associations of paternal or maternal support behaviours with children meeting MVPA recommendations (see Additional file 2, Tables S5 and S6) were similar to findings in the original analyses.

## Discussion

Our study examined the relationships of paternal and maternal support behaviours for PA with the proportions of children meeting the MVPA recommendations on weekdays and weekends in China. We found that the paternal and maternal total scores for support behaviours were associated with higher odds of children meeting the MVPA recommendations on weekends, but not on weekdays. Maternal cultivation of the child’s PA habits was positively associated with children meeting the MVPA recommendations on weekends. Maternal reservation of PA time for the child was positively associated with children meeting MVPA recommendations on weekdays and weekends. Paternal PA knowledge-sharing with the child was positively associated with children meeting MVPA recommendations on weekdays and weekends.

Our study found that the paternal and maternal total scores of support behaviours for PA were positively associated with higher odds of children meeting the MVPA recommendations on weekends. Our results are consistent with a previous report that family support promotes MVPA in adolescents [37]. For each unit increase in family support, the daily weekday MVPA increased by 0.86 min/day, while the weekend MVPA increased by 1.28 min/day [37]. The positive association between parental support for PA and their child’s increased MVPA might be mediated by their child’s increased self-efficacy [38]. A systematic review showed that adolescents’ self-efficacy had consist and positive associations with physical activity in Chinese studies [39]. There are more PA opportunities within school settings on weekdays, such as PE class, and school-level PA support works more on children’s MVPA [40, 41]. In contrast, children’s activities on weekends are likely to be at home or in the community. In these settings, parents could support their children by caring about staying fit and exercising, encouraging their children to be physically active, and being active with their children [29]. A previous study also showed that increased parental encouragement was positively associated with PA among girls on weekend days, while increased parental care was associated with PA among boys on weekend days [29]. Weekends might be the optimal time for parents to support their child’s MVPA. Our study revealed that only 37.8% of children met the MVPA recommendations on weekends, which was significantly lower than the proportion on weekdays. This result is consistent with an undesirable global trend [13, 42]: a meta-analysis showed that children were less active on weekends than on weekdays [13], while the reported proportions of adolescents meeting the MVPA recommendations were 17% on weekends and 31% on weekdays [29, 42].

Our study indicated that a higher odds of children meeting the MVPA recommendations was positively associated with maternal cultivation of PA habits and reservation of PA time for the child; it was not associated with maternal PA knowledge-sharing with the child. Our findings are supported by evidence that maternal encouragement may not always promote children’s PA [43]. Some mothers may share PA knowledge with their children by nagging [44], which might be considered bothersome and negatively influence the child’s MVPA [45]. In contrast, our study suggested that direct support behaviours, such as cultivating children’s PA habits and reserving PA time for children, are positive venues for mothers to guide children in performing MVPA. Similarly, Tanaka et al. reported that children whose mothers provide direct support (e.g., by watching children’s sporting events) had higher MVPA levels, compared with children whose mothers did not watch the events [26]. Therefore, maternal direct support behaviours may improve MVPA levels among their children.

Our study contributes to the literature that paternal PA knowledge-sharing with the child is positively associated with children’s MVPA. This was similar with that paternal verbal support behaviour (i.e., praising child for participating in physical activity) was positively associated with MVPA after school among child and adolescent boys [27]. However, a Japanese study failed to find significant associations between paternal support (e.g., by engaging in PA with their child on weekdays and weekends, encouraging their children’s PA, or watching children’s sporting events) and children’s MVPA [26]. The specific support behaviours for PA by fathers varied in the above studies, and the associations remained unclear [19]. Studies assessing comprehensive paternal support behaviours (e.g., sharing PA knowledge, encouraging PA, watching children’s PA, modelling PA, and engaging in PA with children) should be conducted to verify the associations between paternal support behaviours for PA and children’s MVPA.

Our study is novel in that it differentiated between paternal and maternal influence on children’s MVPA, while observing children’s MVPA at different times (i.e., weekdays vs. weekends). Paternal and maternal total support scores were both positively associated with children meeting the MVPA recommendations on weekends. The results suggest the importance of paternal and maternal support for children’s MVPA on weekends.

However, our study had some limitations. First, our study was cross-sectional, so causal inferences could not be identified. The pattern of associations may also reflect the possibility that very active children elicited more support from their parents. A longitudinal study is needed to determine the direction of the association between parental PA support and children’s PA. Second, our study included only three aspects of parental support behaviours. Although it had acceptable reliability, the biased response might exist in the second question. Further validation study for the three aspects of parental support behaviours is warrant. In addition, our study did not include other support behaviours, such as parental encouragement of children’s engagement in PA, parents’ engagement in PA with their children PA, or financial and logistic support. Future studies should develop a more comprehensive support behaviour scale. Third, although 7-consecutive-day PA diaries can be used to avoid recall bias and mirror children’s PA patterns over a period of 7 days [46], they are less accurate than an accelerometer. Nevertheless, a PA diary was more feasible than an accelerometer in our study, considering our large sample size. Fourth, our study involved potential selection bias; most of the caregivers’ questionnaires were from mothers (73.3%), which may limit the generalisability of our findings. In addition, in an attempt to reduce respondent burden, only one parent (father or mother) was investigated in our study. The results showed that the different associations of maternal and paternal support behaviours with children’s MVPA might not have been statistically significant in our study. Further studies of both mothers and fathers are needed to confirm the strength of each association.

## Conclusions

Parental total support behaviours for PA have critical roles in promoting children’s MVPA on weekends, implying that appropriate intervention targeting parents may counteract the increasing tendency towards inactivity in children, especially on weekends. Maternal direct support behaviours (e.g.*,* cultivating the child’s PA habits and reserving PA time for the child) and paternal verbal support behaviour (e.g.*,* sharing PA knowledge with the child) influenced MVPA among their children. To improve MVPA in children, tailored intervention strategies targeting specific family support behaviours for PA should be considered separately for fathers and mothers. Longitudinal research should verify the associations of paternal and maternal support behaviours for PA with children’s MVPA on weekdays and weekends.

## Supplementary Information

**Additional file 1.** Physical activity diaries for students.

**Additional file 2: Table S1.** Associations between children’s MVPA time and children’s, paternal and maternal demographic characteristics. **Table S2.** The percentage of children meeting MVPA recommendation among different groups. **Table S3.** Univariate association between paternal support behaviors and children’s MVPA. **Table S4.** Univariate association between maternal support behaviors and children’s MVPA. **Table S5.** Adjusted association of paternal support behaviors and children’s MVPA meeting the recommendation. **Table S6.** Adjusted association of maternal support behaviors and children’s MVPA meeting the recommendation.

## Data Availability

The dataset for the present study is available from the corresponding author upon reasonable request.

## Abbreviations

PA: Physical Activity
MVPA: Moderate-to-Vigorous Physical Activity
PE: Physical Education
OR: Odds Ratio
CI: Confidence Interval
