# Supplementary material for: Paternal and maternal support of moderate-to-vigorous physical activity in children on weekdays and weekends: a cross-sectional study
Source: BMC Public Health. 2021 Sep 30;21:1776. doi: 10.1186/s12889-021-11730-8 (PMC8482694; doi:10.1186/s12889-021-11730-8)
Supplement: Supplementary file 1 — Additional file 1. Physical activity diaries for students. [file 12889_2021_11730_MOESM1_ESM.docx]

**Additional file 1**

**Physical activity diaries for students**

ID (Filled in by researcher): □□□□□□

Please fill in the following information:

Name: _______________

Gender: Male □ Female □

School name: _______________

Grade: _______________

Class: _______________

Start date you filled in this diary: ______Year______Month______Day

End date you filled in this diary: ______Year______Month______Day

**Instructions:**

Starting from today, please record your physical activity close to bedtime every day. Please record for consecutive 7 days.

Please answer the following questions truthfully. Your answers will be kept confidential, and don't hesitate.

**Record methods:**

1 You are required to fill in this diary for 7 days. Please remember to record it close to bedtime every day.

2 Please bring it back to the head teacher after you finished this diary.

3 Please mark "√" in the □ of your answer.

4 If the question requires the number or time to continue to answer, please fill in "_____".

5 Every question is very important, please don't miss it.


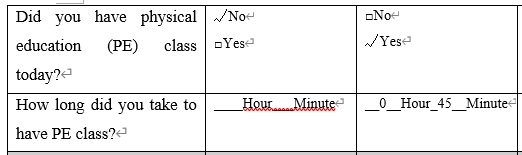


Please record：

|  | the day of the week ____  _____Month_____Day | the day of the week ____  _____Month_____Day | the day of the week ____  _____Month_____Day | the day of the week ____  _____Month_____Day | the day of the week ____  _____Month_____Day | the day of the week ____  _____Month_____Day | the day of the week ____  _____Month_____Day |
| --- | --- | --- | --- | --- | --- | --- | --- |
| How did you go to school? | □ No school  □ Walked  □ Cycled  □ Rode in a car or bus, etc. | □ No school  □ Walked  □ Cycled  □ Rode in a car or bus, etc. | □ No school  □ Walked  □ Cycled  □ Rode in a car or bus, etc. | □ No school  □ Walked  □ Cycled  □ Rode in a car or bus, etc. | □ No school  □ Walked  □ Cycled  □ Rode in a car or bus, etc. | □ No school  □ Walked  □ Cycled  □ Rode in a car or bus, etc. | □ No school  □ Walked  □ Cycled  □ Rode in a car or bus, etc. |
| How long did you take to go to school? | ____Hour___Minute | ___Hour___Minute | ___Hour___Minute | ___Hour___Minute | ____Hour___Minute | ____Hour___Minute | ____Hour___Minute |
| How did you go home after school? | □ No school  □ Walked  □ Cycled  □ Rode in a car or bus, etc. | □ No school  □ Walked  □ Cycled  □ Rode in a car or bus, etc. | □ No school  □ Walked  □ Cycled  □ Rode in a car or bus, etc. | □ No school  □ Walked  □ Cycled  □ Rode in a car or bus, etc. | □ No school  □ Walked  □ Cycled  □ Rode in a car or bus, etc. | □ No school  □ Walked  □ Cycled  □ Rode in a car or bus, etc. | □ No school  □ Walked  □ Cycled  □ Rode in a car or bus, etc. |
| How long did you take to go home after school? | ___Hour___Minute | ___Hour___Minute | ___Hour___Minute | ___Hour___Minute | ___Hour___Minute | ____Hour___Minute | ____Hour___Minute |
| Did you walk outside today?’ (e.g., shopping, visiting relatives or friends, or going to a park) | □No  □Yes | □No  □Yes | □No  □Yes | □No  □Yes | □No  □Yes | □No  □Yes | □No  □Yes |
| How long did you take to walk outside? | ____Hour___Minute | ___Hour___Minute | ___Hour___Minute | ____Hour___Minute | ____Hour___Minute | ____Hour___Minute | ____Hour___Minute |
| Did you do homework today? | □No  □Yes | □No  □Yes | □No  □Yes | □No  □Yes | □No  □Yes | □No  □Yes | □No  □Yes |
| How long did you take to do homework? | ____Hour___Minute | ____Hour___Minute | ____Hour___Minute | ____Hour___Minute | ____Hour___Minute | ____Hour___Minute | ____Hour___Minute |
| Did you watch television today? | □No  □Yes | □No  □Yes | □No  □Yes | □No  □Yes | □No  □Yes | □No  □Yes | □No  □Yes |
| How long did you take to watch television? | ____Hour___Minute | ____Hour___Minute | ____Hour___Minute | ____Hour___Minute | ____Hour___Minute | ____Hour___Minute | ____Hour___Minute |
| Did you play computer/iPad or e-games today? | □No  □Yes | □No  □Yes | □No  □Yes | □No  □Yes | □No  □Yes | □No  □Yes | □No  □Yes |
| How long did you take to play computer or e-games? | ____Hour___Minute | ____Hour___Minute | ____Hour___Minute | ____Hour___Minute | ____Hour___Minute | ____Hour___Minute | ____Hour___Minute |
| Did you have physical education (PE) class today? | □No  □Yes | □No  □Yes | □No  □Yes | □No  □Yes | □No  □Yes | □No  □Yes | □No  □Yes |
| How long did you take to have PE class? | ____Hour___Minute | ____Hour___Minute | ____Hour___Minute | ____Hour___Minute | ____Hour___Minute | ____Hour___Minute | ____Hour___Minute |
| In addition to PE class, did you do moderate-to-vigorous physical activity (MVPA) in school today? MVPA referred to activities that made the child sweat, gasp, or feel a little tired or very tired (e.g., running, playing football, cycling, and dancing) | □No  □Yes | □No  □Yes | □No  □Yes | □No  □Yes | □No  □Yes | □No  □Yes | □No  □Yes |
| How long did you take to do MVPA in school in addition to PE class? | ____Hour___Minute | ____Hour___Minute | ____Hour___Minute | ____Hour___Minute | ____Hour___Minute | ____Hour___Minute | ____Hour___Minute |
| Did you do MVPA outside of school? | □No  □Yes | □No  □Yes | □No  □Yes | □No  □Yes | □No  □Yes | □No  □Yes | □No  □Yes |
| How long did you take to do MVPA outside of school? | ____Hour___Minute | ____Hour___Minute | ____Hour___Minute | ____Hour___Minute | ____Hour___Minute | ____Hour___Minute | ____Hour___Minute |
