# Supplementary material for: Paternal and maternal support of moderate-to-vigorous physical activity in children on weekdays and weekends: a cross-sectional study
Source: BMC Public Health. 2021 Sep 30;21:1776. doi: 10.1186/s12889-021-11730-8 (PMC8482694; doi:10.1186/s12889-021-11730-8)
Supplement: Supplementary file 2 — Additional file 2: Table S1. Associations between children’s MVPA time and children’s, paternal and maternal demographic characteristics. Table S2. The percentage of children meeting MVPA recommendation among different groups. Table S3. Univariate association between paternal support behaviors and children’s MVPA. Table S4. Univariate association between maternal support behaviors and children’s MVPA. Table S5. Adjusted association of paternal support behaviors and children’s MVPA meeting the recommendation. Table S6. Adjusted association of maternal support behaviors and children’s MVPA meeting the recommendation. [file 12889_2021_11730_MOESM2_ESM.docx]

**Additional file 2**

**Table S1** Associations between children’s MVPA time and children’s, paternal and maternal demographic characteristics

|  | **Daily weekday MVPA time** | | **Daily weekend MVPA time** | | **Daily MVPA time** | |
| --- | --- | --- | --- | --- | --- | --- |
|  | ≥60min,n(%) | *χ^2^/P* | ≥60min,n(%) | *χ^2^/P* | ≥60min,n(%) | *χ^2^/P* |
| *Children(n=1939)* |  |  |  |  |  |  |
| Gender |  |  |  |  |  |  |
| Male | 603(62.9) | 0.003/0.955 | 381(39.7) | 3.157/0.076 | 546(56.9) | 0.009/0.926 |
| Female | 615(62.8) |  | 351(35.8) |  | 560(57.1) |  |
| District |  |  |  |  |  |  |
| Urban | 506(56.5) | 28.063/**<0.001** | 344(38.4) | 0.331/0.565 | 462(51.6) | 19.825/**<0.001** |
| Rural | 712(68.2) |  | 388(37.2) |  | 644(61.7) |  |
| Schooling stage |  |  |  |  |  |  |
| Primary | 559(59.9) | 6.786/**0.009** | 405(43.4) | 24.138/**<0.001** | 533(57.1) | 0.001/0.982 |
| Middle | 659(65.6) |  | 327(32.5) |  | 573(57.0) |  |
| Weight status |  |  |  |  |  |  |
| Normal or wasting | 819(64.5) | 4.668/**0.031** | 481(37.9) | 0.036/0.849 | 732(57.7) | 0.621/0.431 |
| Overweight or obesity | 399(59.6) |  | 251(37.5) |  | 374(55.8) |  |
| *Father(n=472)* |  |  |  |  |  |  |
| Age |  |  |  |  |  |  |
| ＜40 years | 178(64.7) | 0.540/0.462 | 109(39.6) | 0.323/0.570 | 168(61.1) | 0.663/0.415 |
| ≥40 years | 121(61.4) |  | 73(37.1) |  | 113(57.4) |  |
| Education level |  |  |  |  |  |  |
| Junior middle school or below | 175(68.9) | 7.301/**0.026** | 99(39.0) | 0.135/0.935 | 164(64.6) | 5.895/0.052 |
| Senior middle school or junior college | 102(57.0) |  | 69(38.5) |  | 97(54.2) |  |
| University or above | 22(56.4) |  | 14(35.9) |  | 20(51.3) |  |
| Weight status |  |  |  |  |  |  |
| Normal or wasting | 117(64.6) | 0.212/0.646 | 70(38.7) | 0.002/0.968 | 110(60.8) | 0.187/0.665 |
| Overweight or obesity | 182(62.5) |  | 112(38.5) |  | 171(58.8) |  |
| *Mother(n=1276)* |  |  |  |  |  |  |
| Age |  |  |  |  |  |  |
| ＜40 years | 594(61.7) | 1.015/0.314 | 374(38.8) | 0.583/0.445 | 542(56.3) | 0.144/0.704 |
| ≥40 years | 203(64.9) |  | 114(36.4) |  | 180(57.5) |  |
| Education level |  |  |  |  |  |  |
| Junior middle school or below | 403(66.6) | 8.588/**0.014** | 215(35.5) | 3.855/0.145 | 361(59.7) | 4.466/0.107 |
| Senior middle school or junior college | 309(59.1) |  | 210(40.2) |  | 281(53.7) |  |
| University or above | 85(57.4) |  | 63(42.6) |  | 80(54.1) |  |
| Weight status |  |  |  |  |  |  |
| Normal or wasting | 425(59.9) | 4.621/**0.032** | 271(38.2) | 0.004/0.950 | 389(54.8) | 2.098/0.148 |
| Overweight or obesity | 372(65.7) |  | 217(38.3) |  | 333(58.8) |  |

**Table S2** The percentage of children meeting MVPA recommendation among different groups

| **Variables** | **All children** (n=1939) | **Children in father-child dyads** (n=517) | **Children in mother-child dyads** (n=1422) |
| --- | --- | --- | --- |
| Daily weekday MVPA≥60min,n(%) | 959(49.5) | 265(51.3) | 694(49.8) |
| Daily weekend MVPA≥60min,n(%) | 704(36.3) | 186(36.0) | 518(36.4) |
| Daily MVPA≥60min,n(%) | 848(43.7) | 236(45.6) | 612(43.0) |

*Note.* The 40.8% of PE times was added to total MVPA minutes. Chi-squared test showed that there was no significant difference of the percentage of children meeting MVPA recommendation between father-child dyads and mother-child dyads. Matched Chi-squared test found significant differences of the percentage of children meeting MVPA recommendation on weekdays (49.5%) and on weekends (36.3%) among all children.

| **Table S3** Univariate association between paternal support behaviors and children’s MVPA | | | | | | |
| --- | --- | --- | --- | --- | --- | --- |
| **Variables** | **Daily weekday MVPA time** | | **Daily weekend MVPA time** | | **Daily MVPA time** | |
|  | ＜60min  (n=252) | ≥60min  (n=265) | ＜60min  (n=331) | ≥60min  (n=186) | ＜60min  (n=281) | ≥60min  (n=236) |
| Total support behaviour scores | 10.15±2.19 | 10.25±2.36 | **10.04±2.28** | **10.49±2.24** | 10.06±2.24 | 10.38±2.31 |
| Share PA knowledge with the child | 3.50±0.89 | 3.52±0.88 | **3.44±0.89** | **3.62±0.87** | 3.47±0.89 | 3.55±0.87 |
| Cultivate the child’s PA habits | 3.48±0.89 | 3.47±0.92 | 3.42±0.90 | 3.57±0.90 | 3.44±0.90 | 3.52±0.90 |
| Reserve PA time for the child | 3.18±0.97 | 3.26±0.99 | 3.18±0.99 | 3.31±0.96 | 3.15±0.98 | 3.31±0.97 |

*Note*. The 40.8% of PE times was added to total MVPA minutes. Bold results refers to significant difference between group ≥60 min and ＜60min in each time interval (*P* <0.05).

| **Table S4** Univariate association between maternal support behaviors and children’s MVPA | | | | | | |
| --- | --- | --- | --- | --- | --- | --- |
| **Variables** | **Daily weekday MVPA time** | | **Daily weekend MVPA time** | | **Daily MVPA time** | |
|  | ＜60min  (n=728) | ≥60min  (n=694) | ＜60min  (n=904) | ≥60min  (n=518) | ＜60min  (n=810) | ≥60min  (n=612) |
| Total support behaviour scores | 9.83±2.14 | 10.04±2.27 | **9.79±2.17** | **10.18±2.25** | 9.83±2.18 | 10.06±2.24 |
| Share PA knowledge with the child | 3.41±0.84 | 3.43±0.87 | 3.40±0.84 | 3.45±0.87 | 3.41±0.84 | 3.43±0.86 |
| Cultivate the child’s PA habits | 3.44±0.84 | 3.47±0.87 | **3.41±0.84** | **3.52±0.87** | 3.44±0.85 | 3.47±0.86 |
| Reserve PA time for the child | **2.99±0.91** | **3.14±0.96** | **2.98±0.93** | **3.20±0.94** | **2.99±0.93** | **3.16±0.94** |

*Note*. The 40.8% of PE times was added to total MVPA minutes. Bold results refers to significant difference between group ≥60 min and ＜60min in each time interval (*P* <0.05).

**Table S5** Adjusted association of paternal support behaviors and children’s MVPA meeting the recommendation

| Variables | **Daily weekday MVPA time** | **Daily weekend MVPA time** | **Daily MVPA time** |
| --- | --- | --- | --- |
| Total support behaviour scores | 1.044(0.961,1.134) | **1.097(1.007,1.195)** | **1.092(1.005,1.186)** |
| Share PA knowledge with the child | 1.146(0.922,1.425) | **1.303(1.039,1.632)** | 1.217(0.980,1.511) |
| Cultivate the child’s PA habits | 1.020(0.825,1.261) | 1.217(0.978,1.513) | 1.146(0.928,1.414) |
| Reserve PA time for the child | 1.114(0.921,1.348) | 1.135(0.934,1.379) | **1.225(1.013,1.481)** |

*Note.* The 40.8% of PE times was added to total MVPA minutes. In each logistic regression, outcome variable is 1=<60min and 2=≥60min. This table provides OR and 95%CI formatted as “OR(lower CI, upper CI)” for each result of logistic regression. Bolded results indicate statistically significant (*P* <0.05). Children’s school stage, gender, district, weight status, and paternal educational level were adjusted in each logistic regression.

**Table S6** Adjusted association of maternal support behaviors and children’s MVPA meeting the recommendation

| Variables | **Daily weekday MVPA time** | **Daily weekend MVPA time** | **Daily MVPA time** |
| --- | --- | --- | --- |
| Total support behaviour scores | 1.052(1.000,1.107) | **1.085(1.029,1.143)** | **1.053(1.000,1.108)** |
| Share PA knowledge with the child | 1.088(0.952,1.243) | **1.083(0.943,1.243)** | 1.073(0.939,1.227) |
| Cultivate the child’s PA habits | 1.045(0.917,1.189) | 1.179(1.030,1.349) | 1.050(0.922,1.196) |
| Reserve PA time for the child | **1.190(1.056,1.341)** | **1.277(1.128,1.445)** | **1.203(1.067,1.356)** |

*Note.* The 40.8% of PE times was added to total MVPA minutes. In each logistic regression, outcome variable is 1=<60min and 2=≥60min. This table provides OR and 95%CI formatted as “OR(lower CI, upper CI)” for each result of logistic regression. Bolded results indicate statistically significant (*P* <0.05). Children’s school stage, gender, district, weight status, and maternal educational level were adjusted in each logistic regression.
